# Seroprevalence, spatial dispersion and factors associated with flavivirus and chikungunya infection in a risk area: a population-based seroprevalence study in Brazil

**DOI:** 10.1186/s12879-020-05611-5

**Published:** 2020-11-24

**Authors:** Francisca Kalline de Almeida Barreto, Carlos Henrique Alencar, Fernanda Montenegro de Carvalho Araújo, Rhaquel de Morais Alves Barbosa Oliveira, John Washington Cavalcante, Daniele Rocha Queiroz Lemos, Luís Arthur Brasil Gadelha Farias, Isac Lucca Frota Boriz, Leticia Queiroz Medeiros, Marcelo Nunes Pereira Melo, Fábio Miyajima, André Machado Siqueira, André Ricardo Ribas Freitas, Luciano Pamplona de Góes Cavalcanti

**Affiliations:** 1 Programa de Pós-graduação em Saúde Coletiva, Universidade Federal do Ceará, Fortaleza, CE Brazil; 2 Programa de Pós-graduação em Patologia, Universidade Federal do Ceará, Fortaleza, CE Brazil; 3 Faculdade de Medicina, Centro Universitário Christus, Fortaleza, CE Brazil; 4 Laboratório Central de Saúde Pública do Ceará, Fortaleza, CE Brazil; 5 Serviço de Verificação de Óbitos Dr Rocha Furtado, Secretaria de Saúde do Estado do Ceará, Fortaleza, CE Brazil; 6 Faculdade de Medicina, Universidade Federal do Ceará, Fortaleza, CE Brazil; 7 Hospital São José de Doenças infecciosas, Fortaleza, CE Brazil; 8 Fundação Oswaldo Cruz Ceará, Eusébio, Brazil; 9 Fundação Oswaldo Cruz, Presidência da Fiocruz, Instituto de Pesquisa Clínica Evandro Chagas (INI/Fiocruz), Rio de Janeiro, Brazil; 10 Faculdade São Leopoldo Mandic, Campinas, SP Brazil

**Keywords:** Seroprevalence, Chikungunya virus, Dengue virus, Zika virus

## Abstract

**Background:**

The State of Ceará, in Northeastern Brazil, suffers from a triple burden of arboviruses (dengue, Zika and chikungunya). We measured the seroprevalence of chikungunya, dengue and Zika and its associated factors in the population of Juazeiro do Norte, Southern Ceará State, Brazil.

**Methods:**

A cross-sectional study of analytical and spatial analysis was performed to estimate the seroprevalence of dengue, Zika and chikungunya, in the year 2018. Participants were tested for IgM and IgG against these three viruses. Those with IgM and/or IgG positive tests results were considered positive. Poisson regression was used to analyze the factors associated with positive cases, in the same way that the spatial analysis of positive cases was performed to verify whether the cases were grouped.

**Results:**

Of the 404 participants, 25.0% (103/404) were positive for CHIKV, 92.0% (373/404) for flavivirus (dengue or Zika) and of these, 37.9% (153/404) samples were classified as probable dengue infection. Of those who reported having had an arbovirus in the past, positive CHIKV cases had 58.7% arthralgia (PR = 4.31; 95% CI: 2.06–9.03; p = 0.000) mainly in the hands, ankles and feet. Age over 60 years had a positive association with cases of flavivirus (PR = 1.29; 95% CI: 1.09–1.54; p = 0.000). Fever, muscle pain, joint pain and skin rash were the most reported symptoms (46.1, 41.0, 38.3 and 28.41%, respectively). The positive cases of chikungunya and dengue or Zika were grouped in space and the city center was most affected area.

**Conclusions:**

Four years after the introduction of CHIKV, where DENV has been in circulation for over 30 years, 1/4 of the population has already been exposed, showing the extent of the epidemic. The measured prevalence was much higher than that reported by local epidemiological surveillance.

## Background

Dengue virus (DENV) has been endemic in the northeastern region of Brazil for over 30 years, with circulation of the four serotypes (DENV 1–4), causing a significant number of cases and several outbreaks in all states of the region [1, 2]. The chikungunya virus (CHIKV) was detected in the Americas in 2014 [3] and caused a major epidemic in 2017 with approximately 190,000 cases reported in Brazil, with 173 deaths, according the SINAN (Portuguese acronym for Notifiable Diseases Information System, official epidemiologic suveilance). Of these, 137,424 (73%) cases were reported in Ceará, Northeast Brazil [3–5]. In 2015, the Zika virus (ZIKV) started to circulate in the Northeast region of Brazil and, in the following years, caused an epidemic with more than 200,000 reported cases. This virus has been associated with many neurological disorders, including congenital syndrome caused by the Zika virus and Guillain-Barré syndrome [6, 7].

*Aedes aegypti* is the main transmitter of these three viruses. They usually cause diseases with very similar clinical characteristics at the beginning, which present themselves as a nonspecific and mild febrile illness, muscle pain, arthralgia, with the addition of a rash, or conjunctivitis / retroorbital pain. This fact can confuse the diagnosis and clinical management of professionals [3, 8].

Juazeiro do Norte is a city located in the south of Ceará, has the 3rd largest population in the state. The city receives approximately 2.5 million people annually due to religious tourism and has a history of dengue transmission for over two decades [9, 10]. Between 2016 and 2017, 1047 cases of chikungunya (two deaths) were reported in the city. Of these, only 173 were laboratory confirmed [5, 7]. In the same period, the incidence of dengue was 778.6/100,000 inhabitants and eight other cases of Zika were reported. Of these, only 106 were laboratory confirmed for DENV and two for ZIKV [4, 11].

Due to passive surveillance in Brazil, only cases cases that seek assistance are confirmed, as a result, it is difficult to estimate the magnitude of the epidemic. In addition, Zika virus and the dengue virus are both flaviviruses and genetically related, which, can cause cross-reaction of serological tests against these viruses [12, 13]. Therefore, establishing a final diagnosis of flavivirus in an endemic area of ZIKV/DENV co-circulation is a challenge, given the similarity of symptoms and the difficulty of serological tests to identify the viruses. Besides, other regional characteristics of Brazil, such as vaccination against the yellow fever virus (YFV), another member of the *Flaviviridae* family, make it difficult to determine the real impact caused by these viruses on Brazilian population [14, 15].

Considering that the state of Ceará had one of the worst chikungunya epidemics in Brazil, with an incidence of 1460.6 cases / 100,000 inhabitants and 245 confirmed deaths in 2016/2017 [5, 11] and that there is a lack of quality information on the real numbers of Zika and dengue infections (the latter circulating for more than 30 years in the region) [9], the aim of this study was to estimate the seroprevalence of chikungunya and dengue or Zika in the population of Juazeiro do Norte, Brazil. In addition, we aim to describe and analyze the epidemiological profile and clinical manifestations associated with serological positivity by viruses and to identify the spatial distribution patterns of positive cases due to chikungunya and dengue or Zika.

## Methods

### Design and study site

**Fig. 1:**
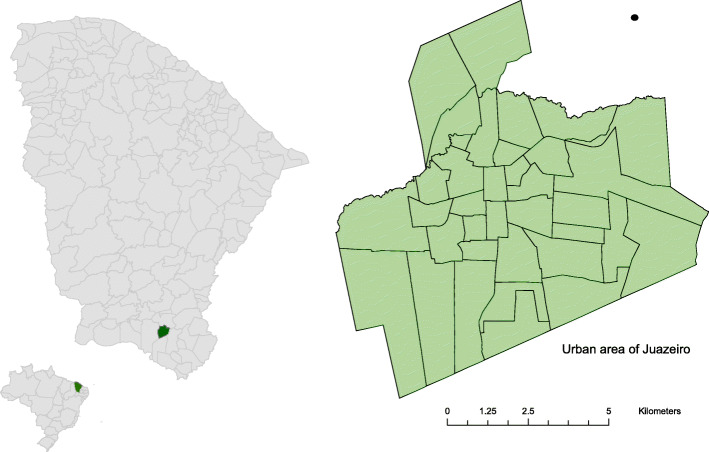
Location of the city of Juazeiro do Norte, Ceará, Brazil

This was an analytical and spatial analysis cross-sectional survey study with data collected between June and December 2018 in the city of Juazeiro do Norte, Northeastern Brazil. The municipality has an estimated population of 271,926 inhabitants, distributed in 37 neighborhoods [10] (Fig. 1).

### Sampling for data collection

Random points were proportionally drawn using the ArcGIS 9.2 software to the population of each neighborhood to identify the collection sites. These points were distributed under the cartographic base of the city, provided by the Brazilian Institute of Geography and Statistics (IBGE); subsequently, all residents of selected house were invited to participate in the study and those who accepted were included. If the point drawn was not a residence or if none resident from the selected residence agreed to participate in the survey, the residence closest to the selected location was selected. In neighborhoods with a population of less than 1000 inhabitants, a minimum of three residences were previously defined.

Sample size was calculated by estimating a prevalence of 50%, with a sampling error of 5% and a confidence level of 95%, using Epi Info 7.2 software, resulting in a minimum sample of 384 individuals.

### Household visit procedures

Houeshold visits were conducted by a trained team, coordinated by a nurse. One venous blood (5 ml) sample was collected per each participant for serological diagnosis and a semi-structured questionnaire was administered. The questionnaire was developed specifically for this study and addressed questions related to sociodemographic factors (age, gender, self-reported skin color, educational level, occupation/work, family income, number of individuals in the household), medical history (cardiovascular diseases, diabetes, hypertension and previous arbovirus diagnosis during pregnancies), household environmental characteristics (type of habitation, water storage, basic sanitation, regular garbage collection, number of toilets, running water) and mosquito control related behaviors (use of mosquito nets or insect repellent for insect protection, and prior knowledge regarding arboviruses).

### Laboratory diagnosis

For serum separation, the collected blood samples were transported to the local laboratory where they were centrifuged under 3000 rpm for 10 min in an EVLAB apparatus (Macro EV model; 04). Subsequently, they were frozen at − 20 °C and later transported to the Central Laboratory of Public Health of Ceará (LACEN-CE) for the tests.

The samples were tested for dengue, chikungunya and Zika using the method of Enzyme-Linked Immunosorbent Assay (ELISA). All samples were tested for IgG (immunoglobulin G) and IgM (immunoglobulin M): to CHIKV detection was performed using the Euroimmun® kits (Lübeck, Schleswig-Holstein, Germany). DENV IgM and IgG detection was performed using the Panbio® kit (Suwon city, Kyonggi province, Korea). Zika IgM detection was performed using the Novagnostic® (Siemens, Berlim, Germany) kits and IgG using the Euroimmun® kits. All tests were conducted according to manufacturers’ instructions.

Samples that showed inconclusive results were retested. All samples with IgM reagents were tested by reverse transcription polymerase chain reaction (RT-PCR) to investigate possible recent infection or co-detection (Table 4 in Appendix).

### Case definition and symptomatic individuals

A case of CHIKV infection were defined as a case of an individual with serum CHIKV IgM and/or IgG antibodies detected using the enzyme-linked immunosorbent assay method.

A case of flavivirus infection was defined as an individual who presented dengue and/or Zika positive serologies (IgM or IgG) detected using the ELISA (excluding indefinite cases).

Probable dengue cases were defined as an individual with positive serology for dengue (IgM or IgG), detected by the ELISA, and negative for Zika (IgM and IgG), excluding undefined cases.

Probable Zika cases was defined as an individual with positive Zika serology (IgM or IgG), detected by the ELISA, and negative for dengue (IgM and IgG), excluding undefined cases.

A case of arbovirus infection was defined as an individual who presented dengue and/or Zika and/or chikungunya positive serologies (IgM or IgG) detected using the ELISA (excluding indefinite cases).

To calculate the proportion of symptomatic persons, we considered those who declared to have had any of these diseases (with medical confirmation or not), which showed suggestive symptoms for these three arbovirus disease (body pain, joint pain, fever, conjunctivitis, rash), between 2016 and 2018; and those who had IgM or IgG positive tests. We considered asymptomatic those who reported they had not any infection but who had positive results for IgM, IgG, or both.

### Data analysis

Data were entered into the Epi Info 7.2 software and analyzed using Stata 15.1 software (Stata Corp LP, College Station, TX, USA).

Absolute and relative frequencies were calculated, and the chi-squared test was used to assess the association associations between seropositive people and sociodemographic and clinical data. The magnitude of these associations was calculated through the crude prevalence ratio (PR).

To adjust for confounding factors, all variables with *p* < 0.20 in the bivariate analysis were adjusted by age group (model 1) by robust Poisson regression. After this first adjustment, the variables that were associated with the outcome were included in robust Poisson’s regression model (model 2). For this last stage of analysis, the variables were categorized hierarchically, according to the class to which they belonged: group 1, socioeconomic and knowledge regarding arboviruses, and group 2, clinical features [16, 17]. All variables with *p* < 0.10 in these latter models were selected as inputs to a final robust Poisson’s regression model to identify significant associations (*p* < 0.05).

To calculate the underreporting of cases, we multiplied the seroprevalence of CHIKV found in the study, by the population of the municipality, to estimate the number of cases that should have been reported as suspect. This number was later divided by the number of cases captured by local surveillance.

### Spatial analysis

The latitude and longitude of each participant residence was marked using a GPS device (Garmin model, etrex30®).

For spatial analysis, the ArcGis 9.2 software was used. Kernel density analysis was used to map CHIKV seropositive and DENV/ZIKV density within the geographic limits of the study region.

Complementary to this analysis, the nearest neighbor method was used [18], which allowed to measure the distance between each positive point and the location of its nearest neighbor, identifying if the distribution of chikungunya cases exhibited clustering or dispersion in space.

Finally, the prevalence coefficient of each neighborhood was calculated by summing the total number of positive cases and dividing this value by the sample of each neighborhood and multiplying this result by 100 [19].

## Results

### Characteristics of the population

Of the 412 volunteers recruited, eight were excluded due to hemolysis of the collected blood, therefore 404 (98.1%) were included in the analysis. More women were included (68.3%) in this study, the median age was of 45 years old (5–91), the most common self-reported ethnicity was brown (or mixed race) (65.1%), low or no education was present in 57.9%, and average family income was below one minimum wage ($954.00 [1US$ = ± $4.00]). All households had a water supply and 97.7% had access to sewage networks. More than half of the participants (54.0%) stored water at home. Most of the population (77.0%) reported knowing the main form of transmission of arboviruses, citing the vector sting and only 2 people mentioned the possibility of vertical or person-to-person transmission (in the case of ZIKV). 72.8% of the participants reported knowing how to avoid arboviruses, mainly citing the correct handling of standing water and waste. A total of 27.7% of the participants reported that they had suffered previous dengue episodes, 22.8% chikungunya and 5.5% Zika. Moreover, 8.4% of the participants confirmed that they vaccinated against Yellow fever (Table 1).

**Table 1:** Sociodemographic and clinical characteristics of population in the City of Juazeiro do Norte, Brazil, 2018

Variables (N°)	Total	%
**Sex (404)**		
Male	128	31.68
Female	276	68.32
**Self-reported skin color (404)**		
White	94	23.27
Mixed race	263	65.10
Black	47	11.63
**Educational level (404)**		
Illiterate	234	57.92
Elementary School	38	9.41
High School	112	27.72
University graduate	20	4.95
**Civil status (404)**		
Married	184	45.54
Single	143	35.40
Widower	46	11.39
Divorced	31	7.67
**Occupation/work (404)**		
Retired	97	24.01
Informal work	83	20.54
Unemployed	67	16.58
Housewife	65	16.09
Permanent job	56	13.86
Student	36	8.91
**Family income (404)**^a^		
Below R$ 954.00	188	46.53
Between R$ 955.00 and R$ 1999.00	119	29.46
Between R$ 2000.00 and R$ 4599.00	85	21.04
R$ 5000.00 or more	06	1.49
Did not answer	06	1.49
**Age group in years (404)**		
Below 9	03	0.74
Between 9 and 19	41	10.15
Between 20 and 45	177	43.81
Between 46 and 60	69	17.08
60 or more	114	28.22
**Underlying diseases (404)**		
Hypertension	104	25.74
Diabetes	50	12.38
Cholesterol	47	11.63
Anxiety	20	4.95
Arthritis	18	4.46
Depression	13	3.22

Subtitle: ^a^ R$4,00 = 1 U$$;

### Seroprevalence of chikungunya infection and its associated factors

Almost one-quarter (25.5%) of the samples were positive for chikungunya. Of these, five (5%) were positive only for IgM, 98 (95%) only for IgG, and five (4.8%) for both.

During the study period, 1047 suspected cases of CHIKV were officially notified by epidemiological surveillance. Based on the seroprevalence found (25.5%) and considering the total population of Juazeiro do Norte (271,926 inhabitants) we should have had approximately 69 thousand reported cases. Therefore, 66 times as many cases that were caught by the health service should have been reported.

Among the 103 participants with reactive samples, 58.3% were symptomatic (at some point during the past 3 years). Among the 92 participants who reported to have had the disease previously, only 68.9% were confirmed by serology. For participants who did not report have symptoms, 43 (14.3%) had reactive antibodies against the virus, and must be classified as asymptomatic or oligosymptomatic cases.

The proportion of seropositivity was higher in males (66.1%), mixed race (64.1%), elementary schooling (56.4%) as the educational level, 20–45-year age group (39.8%), and participants with family monthly income lower than one minimum wage [(48.5%), minimum monthly wage = R$954,00 (U$$238,50), at the time of research], but a statistically significant difference was not observed between the groups (Table 2).

**Table 2:** Sociodemographic factors of seropositive participants for chikungunya, flavivírus and probable dengue from seroepidemiological research in the city of Juazeiro do Norte, Brazil, 2018

Variables (N°)	N	Positive Chikungunya patients			Positive ***Flavivirus*** patients			Probable Dengue Cases		
		N +	Crude PR (95% CI)	PR (95% CI) Adjusted by age group	N +	Crude RP (95% IC)	PR (95% CI) Adjusted by age group	N +	Crude RP (95% IC)	PR (95% CI) Adjusted by age group
**Gender (404)**										
Male	128	68	1.12 (0.75–1.58)	–	120	0.97 (0.92–1.03)	–	46	1.07 (0.81–1.41)	–
Female	276	35	1.00	–	253	1.00	–	107	1.00	–
**Age group in years (404)**										
4–19	44	09	1.00	–	33	1.00	–	21	1.00	–
20–45	177	41	1.13 (0.59–2.15)	–	161	1.21 (1.01–1.44) **	–	68	1.24 (0.86–1.78)	–
46–60	69	15	1.06 (0.63–2.21)	–	68	1.14 (0.94–1.39)	–	27	1.21 (0.79–1.86)	–
> 60	114	38	1.62 (0.86–3.08)	–	111	1.29 (1.09–1.54) **	–	37	1.47 (0.97–2.20)	–
**Educational level (404)**										
Illiterate	234	65	1.85 (0.63–5.36)	–	217	1.03 (0.88–1.19)	–	73	1.92 (1.28–2.88)**	–
Elementary School	38	10	1.75 (0.54–5.65)	–	36	1.05 (0.89–1.24)	–	13	1.75 (0.99–3.09)	–
High School	112	25	1.48 (0.49–4.46)	–	102	1.01 (0.86–1.18)	–	55	1.22 (0.81–1.83)	–
University graduate	20	3	1.00	–	18	1.00	–	12	1.00	–
**Occupation/work (404)**										
Retired	97	31	1.17 (0.54–2.55)	–	93	1.19 (1.01–1.41)	–	26	1.07 (0.69–1.65)	–
Informal work	83	19	1.64 (0.79–3.39)	–	54	1.19 (1.01–1.41)	–	23	2.20 (1.29–3.42)**	–
Unemployed	67	18	1.38 (0.63–2.99)	–	79	1.18 (0.99–1.39)	–	30	1.38 (0.89–2.13)	–
Housewife	65	15	1.18 (0.53–2.63)	–	61	1.16 (0.98–1.38)	–	26	1.25 (0.80–1.94)	–
Permanent job	56	13	1.19 (0.52–2.70)	–	57	1.05 (0.87–1.27)	–	30	1.11 (0.73–1.70)	–
Student	36	07	1.00	–	29	1.00	–	18	1.00	–
**Family income (404)** ^**£**^										
< 954	188	50	1.59 (0.26–9.70)	–	175	1.11 (0.77–1.60)	–	68	1.08 (0.34–3.41)	–
Mid 954 and 1999	119	31	1.56 (0.25–9.46)	–	108	1.08 (0.75–1.56)	–	42	1.05 (0.33–3.36)	–
Mid 2000 and 4599	85	19	1.34 (0.21–8.38)	–	80	1.12 (0.78–1.62)	–	38	1.34 (0.42–4.26)	–
≥ 5000	06	01	1.00	–	05	1.00	–	02	1.00	–
**Self-reported arbovirus**										
CHIKV	92	64	5.05 (3.70–6.89)**	4.99 (3.64–6.84)**	88	1.03 (0.94–1.14)	–	27	1.21 (0.75–1.95)	–
DENV	112	29	1.01 (0.70–1.46)	2.37 (1.32–4.25)**	108	1.06 (1.00–1.11)	1.05 (1.01–1.11)*	47	1.15 (0.88–1.50)	–
ZIKV	22	10	1.85 (1.13–3.03)*	–	21	1.04 (0.99–1.10)	–	10	0.79 (0.56–1.10)	–
**Underlying diseases**										
Hypertension	104	32	1.33 (0.92–1.93)	–	102	1.09 (1.04–1.15)**	1.00 (0.98–1.03)	28	1.70 (1.20–2.40)**	1.05 (1.02–1.07)**
Diabetes	50	15	1.26 (0.78–2.03)	–	50	1.11 (1.06–1.15)**	1.01 (0.99–1.03)	17	1.27 (0.84–1.92)	0.83 (0.55–1.27)
High Cholesterol	47	15	1.28 (0.81–2.00)	–	47	1.09 (1.06–1.13)*	1.04 (1.01–1.07)**	14	0.76 (0.48–1.20)	0.82 (0.51–1.32)
Anxiety	20	7	1.39 (0.74–2.59)	–	20	1.08 (1.05–1.12)*	1.06 (1.02–1.09)**	08	1.05 (0.61–1.83)	1.10 (0.62–1.95)
Asthma	8	5	2.51 (1.43–4.41)**	2.33 (1.31–4.11)**	7	1.08 (1.05–1.10)	–	7	1.08 (1.05–1.10)	–
Artrhitis	18	9	2.04 (1.24–3.34)**	1.78 (1.05–3.00)**	8	1.08 (1.04–1.11)	–	8	1.08 (1.04–1.11)	–
**Care of standing water and garbage (404)**	**242**	**55**	**0.76 (0.54–1.06)**	**–**	**224**	**1.00 (0.94–1.06)**	**–**	**103**	1.37 (1.04–1.81)**	0.90 (0.85–0.95)**

Subtitle: *N* total number. *N+* number of positive pacients. *PR* Prevalence ratio. ^*^0.01 < *p* < 0.05 and ^**^p < 0.01

After adjusting for age, the following variables were still associated with CHIKV serological diagnosis: accumulating water in drums (PR = 1.43; 95% CI: 1.02–2.01; *p* = 0.034), having asthma (PR = 2.32; 95% CI: 1.31–4.11; *p* = 0.004) and arthritis (PR = 1.77; 95% CI: 1.05–2.99; *p* = 0.011). However, in model 2, by adding the socioeconomic variables, only asthma was still associated with CHIKV serological diagnosis (PR = 1.81; 95% CI: 1.03–3.17; *p* = 0.024).

### Seroprevalence of flavivirus infection and its associated factors

More than 92% (373/404) of the samples were positive for flavivirus, of these 370 were positive for DENV (9 IgM DENV and 370 IgG) and 216 for ZIKV (9 IgM and 216 IgG for ZIKV). None of the patient was considered to be acute due to the absence of positivity only for IgM. Of the people who reported the previous disease due to some flavivirus, 28.9% reported dengue and 5.6% Zika.

The proportion of seropositivity was higher in females (67.8%), primary education (62.7%) and family income below one minimum wage (46.9%), but with no statistically significant difference between groups (Table 2). Older age groups were significantly associated with previous exposure to flavivirus, this variable was more prevalent between 20 and 45 years old (43.1%) (PR = 1.21; 95% CI: 1.01–1.44; *p* = 0.000), and those over 60 years old (29.8%) (PR = 1.29; 95% CI: 1.09–1.54; p = 0.000).

### Seroprevalence of dengue and Zika infection and its associated factors

Only 37,9% (153/404) of samples were classified as probable dengue and 0.74% (3/404) as probable Zika. Probable dengue patients were predominantly female (70%) and 44.4% had family income below one minimum wage. The predominant age group was between 20 and 45 years old (44.4%). 47.7% of the people classified as probable dengue were illiterate with a higher prevalence of positivity (PR = 1.92, 95% CI: 1.28–2.88, *p* = 0.002). Retired people were also associated with greater positivity for these cases, with a prevalence ratio 2.10 higher than non-cases (95% CI: 1.29–3.42; *p* = 0.017). The other variables associated with the outcome were: having systemic arterial hypertension (PR = 1.03; 95% CI: 1.01–1.06; p = 0.002) and knowing that care such as standing water and garbage are protective factors against the proliferation of the vector (PR = 0.92; 95% CI: 0.87–0.97; *p* = 0.007) (Table 2).

### Seroprevalence of arbovirus infection and its associated factors

It is important to note that 92.82% (375/404) of the patients obtained positive serology for at least one of the three types of virus. Of these, 63 (16,8%) patients were IgG positive for the three viruses (DENV, ZIKV, CHIKV), 36 patients were positive only for dengue and chikungunya, and no patient was positive for only Zika and chikungunya.

When corrected for age and multivariate regression, only cholesterol was associated with seropositivity for the presence of the three viruses (PR = 1.04; 95% CI: 1.01–1.07; *p* = 0.008) and having reported the presence of previous chikungunya (PR = 1.06; 95% CI: 1.01–1.11; *p* = 0.006).

### Clinical manifestations of participants with any symptom of arbovirus in the previous three years

The most prevalent symptom for CHIKV was arthralgia, which was present in 60.2% of cases (62/103). This type of arthralgia mainly affected the wrists (24%), fingers (26%), knees and ankles (20%), and feet (17%); polyarticular complaints were frequent (24%). The pain was classified as severe in 59.7% of the cases reporting arthralgia, and it was chronic in 41.7% of these cases (at the time of data collection). Morning stiffness was present in 43.7% of cases, which persisted for more than 20 days. Fever was the second most reported symptom among the participants (56.3%) (58/103), with a mean duration of 6.3 days (PR = 0.66; 95% CI: 0.42–1.03; *p* = 0.08).

After the adjustment by age, with Poisson regression, the associated symptoms in the positive cases of chikungunya were joint pain (PR = 4.31; 95% CI: 2.06–9.03; *p* < 0.001) and back pain (PR = 0.35; 95% CI: 0.18–0.66; *p* < 0.001) (Table 3).

**Table 3:** Signs and symptoms associated with CHIKV and flavivirus infection, in the last three years, in the seroepidemiological survey in the City of Juazeiro do Norte, Brazil, 2018

Signs and symptoms	Chikungunya			Flavivirus		
	PR Crude (95%CI)	PR (95% IC) Adjusted by age group	PR (95%CI) Adjusted	PR Crude (95%CI)	PR (95% IC) Adjusted by age group	PR (95%CI) Adjusted
**Joint pain**	3.85 (1.79–8.24)	3.75 (1.76–8.02)*	4.31 (2.06–9.03)**	1.07 (1.02–1.13)	1.06 (1.01–1.12)**	–
**Fever**	0.61 (0.39–0.94)	0.66 (0.42–1.03)	–	1.05 (0.99–1.11)	1.05 (1.00–1.11) **	–
**Back pain**	0.41 (0.21–0.78)**	0.41 (0.21–0.77)**	0.35 (0.18–0.66)**	1.00 (0.91–1.09)	–	–
**Edema**	1.04 (0.78–1.39)	–	–	–	–	–
**Rash**	1.03 (0.74–1.44)	–	–	1.07 (1.02–1.12)	1.07 (1.02–1.13) **	1.07 (1.02–1.13)**
**Myalgia**	0.73 (0.50–1.05)	0.72 (0.50–1.05)	–	–	–	–
**Headache**	1.32 (0.95–1.83)	1.32 (0.95–1.82)	–	1.02 (0.95–1.08)	–	–
**Abdominal pain**	1.25 (0.90–1.74)	1.27 (0.91–1.77)	–	1.05 (1.00–1.11)	1.06 (1.01–1.12) *	–
**Retroorbital pain**	0.69 (0.46–1.03)	0.68 (0.46–1.02)	0.71 (0.50–1.03)	1.05 (1.00–1.11)	1.05 (0.99–1.11)	–
**Alopecia**^**#**^	1.20 (0.87–1.65)	–	–	–	–	–
**Morning stiffness** ^**#**^	1.26 (0.91–1.74)	–	–	–	–	–
**Muscle pain**	–	–	–	1.06 (1.01–1.11)	1.05 (1.01–1.11) *	–

Subtitle: *PR* Prevalence Ratio; ^*^ *p*-value between 0.01 and 0.05 and ^**^p-values< 0.01

The most prevalent symptoms for flavivirus cases by those who previously reported any of these viruses were fever (46.1%) with an average duration of 6 days (PR = 1.05; 95% CI: 1.00–1.11; *p* = 0.044), the muscle pain was present in 41.0% of cases (PR = 1.05; 95% CI: 1.01–1.11; *p* = 0.033), joint pain in 38.3% (PR = 1.06; 95% CI: 1.01–1.12; *p* = 0.008) and rash in 28.41% (Table 2). Of these, the only one that remained associated in the final explanatory model was the presence of a rash with PR = 1.07 (95% CI: 1.02–1.13; *p* = 0.003), the same with positivity for arbovirus (PR = 1.08; 95% CI: 1.01–1.14; *p* = 0.014). No symptoms previously described were associated with positivity for probable dengue.

### Spatial analysis

Analysis of the nearest neighbor indicated that CHIKV-positive serologies were grouped in space, with an index of 0.531 (*p* < 0.001). Kernel analysis (Fig. 1A) showed the central area of the city as the most affected area. Some points were located in the peripheral areas, identifying low-density areas in the northwest and south regions of Juazeiro do Norte.

**Fig. 2:**
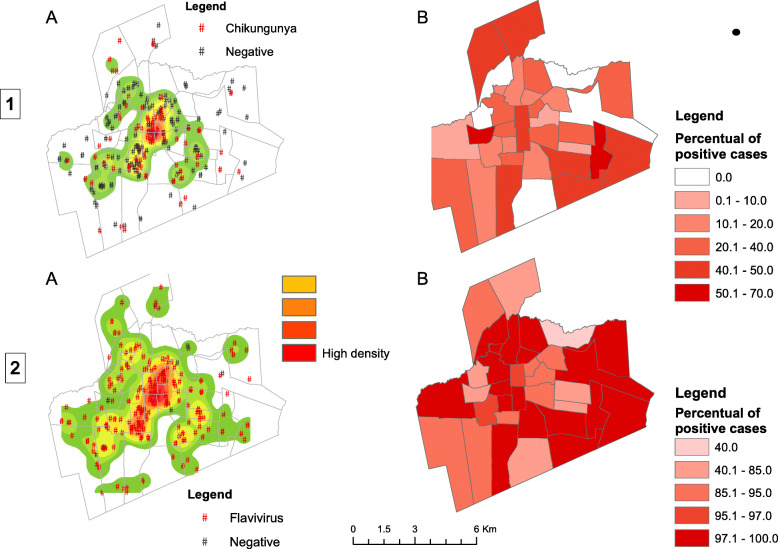
Kernel density (**a**) and seroprevalence (**b**) of chikungunya-positive (1) and flavivirus-positive (2) cases from the seroepidemiological survey in the City of Juazeiro do Norte, Brazil, 2018

The distribution of the prevalence of CHIKV-positive cases (Figs. 1 and 2B) showed that some peripheral neighborhoods had high seroprevalence of infection, with a percentage of cases ranging from 40 to 70%, and others have shown a low or zero prevalence rate, showing that the disease has spread heterogeneously. It is noted a high seroprevalence of cases in the southeast region of the city in three neighborhoods and another in the northern region in one neighborhood.

To flavivirus, the nearest neighbor analysis indicated that positive serologies are grouped in space, with an index of 0.275 (*p* < 0.001). The kernel density analysis (Fig. 2A) showed the central area of the municipality as the most affected. However, in all neighborhoods, positive people were found for flavivirus. Regarding the prevalence analysis, no region had a prevalence below 40%, reaching 100% in some regions (Fig. 2B).

## Discussion

To out knowledge, this is the first seroepidemiological survey carried out in the state of Ceará, which has an arbovirus incidence of 410,0/100,000 inhabitants [11]. We found a 25.5% positivity for CHIKV with a strong association with the presence of arthralgia. The prevalence was much higher for exposure to flavivirus (90%) probably due to the prolonged exposure of the population to the dengue virus [1]. These findings are very different when compared to the numbers reported to SINAN between the years 2016 and 2017: 1047 cases of chikungunya were reported, but only 173 were laboratory confirmed, 2135 cases of dengue with 106 confirmed and 08 of Zika, with only 02 confirmed [11]. This reinforces the importance of conducting serological and longitudinal research to estimative true incidence rate.

The seroprevalence of CHIKV found in this study was lower compared to Feira de Santana (57.1%) and higher than the one of Riachão do Jacuípe (20%), other smaller cities in Northeast, Brazil [20, 21]. Worldwide, a wide range of seroprevalences after first epidemic was observed between 12 and 76% [22–33]. Probably this wide variation is associated with the local climatic differences and the specific characteristics of the evaluated samples. The virus has been known to circulate longer in African and Asian countries, justifying the higher prevalence of CHIKV-positive cases in these countries [3, 24]. In the Americas, CHIKV was detected in late 2013 and the high prevalence of cases observed in some studies is due to an appropriate climate for the presence and dispersion of the vector and the susceptibility of people to the virus [3].

In this study, the prevalence of the people who did not related any symptom, in the last 3 years, and was positive to antibodies against CHIKV was less than 15%, this characterizes the asymptomatic cases. Similar value was observed in French Polynesia after the 2014 outbreak and there is a large variation among reported asymptomatic patients ranging from 4 to 82% [25]. This contradicts the assumption that chikungunya is a disease with a consistent clinical presentation, and the absence of symptoms may be due to the genetic and immunological factors of the affected individuals and the virus specificities [25, 26]. Furthermore, it is important to emphasize that, even if the population was previously exposed to DENV, the possibility of cross-reaction is much less, since it is an *Alphavirus* and despite the enzootic transmission of the Mayaro virus in the Brazilian Amazon, there is no presence of this *Alphavirus* in the study area [27].

In Ceará state, DENV has been circulating since the 1980s [28], and a survey conducted in the city of Fortaleza in 1998 found a prevalence of 44%. Increasingly prevalence has been found over the years in large Brazilian cities such as São Luís in the state of Maranhão (41%), Salvador- capital of the state of Bahia (69%), in Brasiléia and Epitacolância- cities in the state of Acre - North region of Brazil (60.3 to 67.2%) and Recife- capital of the state of Pernambuco (74 to 91%) [28–32].

Worldwide, extensive variations in the seroprevalence of *Flaviviruses* are reported, the lowest are in African countries (between 12 and 50%) and Asia (25%). Among Latin America countries the prevalence reaches approximately 70% [25, 33–38]. On the other hand, the prevalence of ZIKV it is more prominent where it caused major epidemics, such as in French Polynesia (66%) and Yap Islands (73%) [25, 26]. This high prevalence may be attributed to several factors, including ineffectiveness of the *Aedes aegypti* control program, population growth, greater urbanization and climate change, environmental characteristics, mobility degree, among other factors [33, 37].

This study also found a prevalence of almost 40% of probable cases of dengue and less than 1% as probable Zika. This low detection of isolated antibodies may be due to cross reactions between these two viruses. There is a lot of discussion about the cross reactions between flaviviruses, such as DENV, ZIKV or YFV. There are hypotheses that previous infection by a flavivirus may provide protection against subsequent infections by viruses from the same family [14] or even generate an antibody dependent enhancement (ADE), caused by the cross-reaction between genetically similar viruses, such as DENV and ZIKV [39]. Based on the ADE hypothesis, after a previous DENV infection in an individual, a subsequent ZIKV infection can be considered as a second infection and lead to an elevated early IgG response against ZIKV. This may cause a low or absent IgM ZIKV response, so subsequent infections may be more serious due to these past immune reactions [12, 40]. However, until now, none of the hypotheses has been confirmed or ruled out. So, it remains to work with the local reality, which is the probability of cross-reaction of tests based on searches for antibodies, generating presumptive infections [38].

The low detection of ZIKV can be explained by these mechanisms mentioned above. In Brazil and worldwide, the detection of ZIKV has been a problem as it is difficult to estimate the real number of people affected by the virus, due to the similarity between the symptoms and the possibility of cross-reaction between the tests [12].

No associations were found between positive cases for chikungunya and factors such as sex, age, or race, a pattern already suggested in other studies [21, 22]. However, the age was an important factor associated with exposure to flavivirus and it is known as an important characteristic related to the exposure to these diseases, because the older the person is, the greater the probability of being exposed to any of these viruses in their lifetime [25, 32, 34, 38]. Though, some studies have reported that children are being exposed exposed earlier and earlier, between 10 and 15 years old [37, 38, 41]. This may indicate how high has been the prevalence of these diseases.

The sociodemographic variables that were associated with a higher prevalence of probable dengue cases were the fact of being retired and also the group of illiterate people. These aspects are confounded, since most retirees in this study were illiterate. This might happen because retirees stay longer in their homes, providing a greater probability of exposure to the vector. Among the possible individual and family risk factors that predict dengue infection, low socioeconomic status is included [27, 32, 42, 43] and less education [44, 45]. Corroborating social vulnerability as a risk factor, we found in this study a high level of misinformation about modes of transmission or prevention of this diseases, especially of the Zika virus, given the low proportion of people who claimed to recognize the possibility of its transmission through sexual contact or vertical [46].

In relation to clinical variables, positive cases of chikungunya reported a prevalence of polyarthralgia 4 times higher than non-cases, this evidence how this symptom has been considered as an important marker of the disease, and self-reported joint pain could be used to establish the diagnosis of the disease. Such association has been observed in several studies, consistently confirming arthralgia as the main symptom of CHIKV infection. The main sites affected by arthralgia are the hands, feet, and ankles [20, 26, 47, 48] and this arthralgia was chronic in more than 40% of cases. The pathogenesis of chronic chikungunya has not yet been fully elucidated. However, there are already some factors potentially associated with the chronic risk, such as age over 40 years, female gender and immunological factors, such as a higher concentration of some specific cytokines during the acute phase [26, 47, 49, 50]. This data is important, since the chronic pain generated by chikungunya considerably reduce the quality of life of those affected, also compromises family income and hence the local economy [20, 21]. We also found that previous arthralgia was a factor associated with the positive cases of chikungunya. This factor may be associated with a worse classification of pain during chikungunya infection and also with chronic cases [48, 49]. Although asthma was a factor associated with positive cases of chikungunya, we found no data to justify this association. In addition, a recent study showed that patients who have asthma do not show worse symptoms when infected with chikungunya [51, 52].

Previous underlying diseases such as hypertension and high cholesterol were more prevalent in people classified as cases of flavivirus. We did not find any data in the literature to justify such an association, but this data may be due to the high prevalence of hypertension in the Brazilian population (around 30%) [53]. The only self-reported symptom which remained associated with previous exposure to flavivirus was rash, which is much more frequent in dengue and Zika cases than other arboviruses such as chikungunya. Some studies have found an association between skin rash and dengue infections [35] and also with ZIKV infection [54].

A point to be emphasized in relation to the results is the self-report of previous infections. 96% (108/112) of the patients who reported having dengue had reactive antibodies, 95.45% (21/22) of the patients who reported having had Zika also had reactive antibodies (remembering that there may be a cross-reaction between these two viruses, so we cannot say with certainty whether it was dengue or Zika in these cases, but the report of the previous disease was sensitive in diagnosing the disease for the patient). Like 69.6% (64/92) of the patients who reported previous chikungunya, had antibodies against CHIKV. Therefore, the referred symptoms can be used as sensitive markers by the population to identify these diseases.

The spatial analysis showed that cases of chikungunya were throughout the city, but they were observed in the most populous regions, which is consistent with a more recent introduction into the Americas. The same was observed with the cases of flavivirus that spread heterogeneously, but were more concentrated in the central regions of the city, where there is strong trade. The large number of individuals circulating in these areas increases population density and predisposes to a greater spread of the disease, as the flow of individuals can serve as a vehicle for vectors and viruses [55, 56]. A study carried out in the city of Rio de Janeiro, a city with a high population density, demonstrated the presence of clusters for DENV and ZIKV, with simultaneous cases, suggesting that exposure to flavivirus was general in the city and and that there may be a predominance of one virus over another [41, 57]. However, it is more likely that viruses circulate concurrently [58].

## Conclusions

The prevalence of chikungunya was 25% in the population of Juazeiro do Norte, in 2018, with severe arthralgia (60%) being a major factor in the recognition of the disease by patients (PR = 4.75). Such cases tend to become chronic (40%), which negatively impacts on the patient’s life and also the public health system. These findings of this study indicated that the magnitude of the outbreak of CHIKV was significantly greater (66 times) than that reported by the health service in the City of Juazeiro do Norte, based on the number of cases reported through passive surveillance used in Brazil. This low herd immunity maintains the risk of a new chikungunya epidemic for the next few years. The prevalence of exposure to flavivirus was 92%, with age being the factor most associated with exposure to these diseases. It is important to note that the self-report of previous arboviruses obtained an important association with the reactive cases by serology, and can be used as an important marker. The high prevalence of these viruses, especially dengue, shows that arboviruses are a problem and means must be found for better control and classification of cases of these diseases, since the number of reported cases was much lower than that found in the study.

### Limitations of the study

The study did not use other diagnostic tests to detect antigens such as Plaque Reduction Test to distinguish the specificity of antibodies, therefore we cannot affirm whether the patient was specifically exposed to DENV, ZIKV or both and also the DENV serotypes were not identified. In addition, there may memory bias at the time when people were asked about previous exposures to different viruses. Another important fact is that there was less access to the population in neighborhoods with higher family income than in neighborhoods with lower family income. Therefore, low-income families may be underrepresented. Additionally, the cross-sectional design of the study makes it difficult to accurately assess the temporal association between this exposure and the occurrence of infection.

## Data Availability

The data sets used and/or analyzed during the present study are available with the corresponding author, upon reasonable request.

## Appendix

**Table 4:** Reverse transcription polymerase chain reaction for chikungunya virus of the seroepidemiological survey participants in the City of Juazeiro do Norte, Ceará, 2018

Sample N°	RT-PCR	IgG	IgM
**14**	Negative	(+)	(+)
**22**	Negative	(−)	(+)
**29**	Positive	(−)	(+)
**47**	Negative	(+)	Inc
**48**	Positive	(+)	(+)
**76**	Negative	(+)	Inc
**135**	Positive	(+)	Inc
**187**	Negative	(−)	(+)
**213**	Negative	(+)	(+)

Subtitle: (+)  reactive, (−) nonreactive, *Inc.* Inconclusive

## Abbreviations

ADE: Antibody dependent enhancement
CHIKV: Chikungunya virus
CI: Confidence interval
DENV: Dengue virus
ELISA: Enzyme-Linked Immunosorbent Assay
IgG: Immunoglobulin G
IgM: Immunoglobulin M
IBGE: Brazilian Institute of Geography and Statistics
LACEN-CE: Central Public Health Laboratories of Ceará
PR: Prevalence ratio
RT-PCR: Reverse transcription polymerase chain reaction
YFV: Yellow fever virus
ZIKV: Zika virus
